# Temperature Range Shifts for Three European Tree Species over the Last 10,000 Years

**DOI:** 10.3389/fpls.2016.01581

**Published:** 2016-10-25

**Authors:** Rachid Cheddadi, Miguel B. Araújo, Luigi Maiorano, Mary Edwards, Antoine Guisan, Matthieu Carré, Manuel Chevalier, Peter B. Pearman

**Affiliations:** ^1^Centre Nationnal de la Recherche Scientifique, Institut des Sciences de l'Evolution, University Montpellier IIMontpellier, France; ^2^Departamento de Biogeografía y Cambio Global, Museo Nacional de Ciencias Naturales, CSICMadrid, Spain; ^3^Center for Macroecology, Evolution and Climate, Natural History Museum of Denmark, University of CopenhagenCopenhagen, Denmark; ^4^InBIO-CIBIO, University of ÉvoraÉvora, Portugal; ^5^Dipartimento di Biologia e Biotecnologie “Charles Darwin, ” Università di Roma “La Sapienza”Roma, Italy; ^6^Geography and Environment, University of SouthamptonSouthampton, UK; ^7^College of Natural Science and Mathematics, University of AlaskaFairbanks, AK, USA; ^8^Department of Ecology and Evolution, University of LausanneLausanne, Switzerland; ^9^Institute of Earth Science Dynamics, University of LausanneLausanne, Switzerland; ^10^Department of Plant Biology and Ecology, Faculty of Sciences and Technology, University of the Basque Country, UPV/EHULeioa, Spain; ^11^IKERBASQUE, Basque Foundation for ScienceBilbao, Spain

**Keywords:** Holocene, past climate reconstruction, niche conservatism, *Abies*, *Fagus*, *Picea*

## Abstract

We quantified the degree to which the relationship between the geographic distribution of three major European tree species, *Abies alba, Fagus sylvatica* and *Picea abies* and January temperature (Tjan) has remained stable over the past 10,000 years. We used an extended data-set of fossil pollen records over Europe to reconstruct spatial variation in Tjan values for each 1000-year time slice between 10,000 and 3000 years BP (before present). We evaluated the relationships between the occurrences of the three species at each time slice and the spatially interpolated Tjan values, and compared these to their modern temperature ranges. Our results reveal that *F. sylvatica* and *P. abies* experienced Tjan ranges during the Holocene that differ from those of the present, while *A. alba* occurred over a Tjan range that is comparable to its modern one. Our data suggest the need for re-evaluation of the assumption of stable climate tolerances at a scale of several thousand years. The temperature range instability in our observed data independently validates similar results based exclusively on modeled Holocene temperatures. Our study complements previous studies that used modeled data by identifying variation in frequencies of occurrence of populations within the limits of suitable climate. However, substantial changes that were observed in the realized thermal niches over the Holocene tend to suggest that predicting future species distributions should not solely be based on modern realized niches, and needs to account for the past variation in the climate variables that drive species ranges.

## Introduction

Changes in climate may cause shifts in the geographic distribution of species (Parmesan and Yohe, 2003; Root et al., 2003), increase extinction rates (e.g., Thuiller et al., 2005), and alter provision of ecosystem services (Schröter et al., 2005). Adaptation of human societies to these global changes requires accurate predictions of the future potential distributions of key species, such as endemic forest trees. Prediction of potential distributions of trees generally involves developing models of the relationship between current climate, distribution, and range dynamics (Guisan and Zimmermann, 2000; García-Valdés et al., 2013), then applying these models to data derived from models of potential future climate. These predictions rely on key aspects of the species climate requirements. In this context, one assumption for predicting species distributions, using niche-based models, is that species occupy all or most suitable geographic areas (Svenning and Skov, 2004). A second necessary assumption for these statistical models is that the species realized environmental niche (Hutchinson, 1957), as determined from empirical observations, remains stable over time, and space, a phenomenon called ecological niche conservatism (Wiens and Graham, 2005; Pearman et al., 2008). The validity of these assumptions remains a matter of contention.

One reason for uncertainty regarding niche conservatism is that species ecological flexibility and/or genetic adaptation can impact the observed degree of conservatism and the accuracy of predicted species distributions. Veloz et al. (2012) find at the generic level that between the late glacial period and today, the realized niches of *Fraxinus, Ostrya/Carpinus*, and *Ulmus* shifted substantially while the niches of other taxa, such as *Quercus, Picea*, and *Pinus strobus*, remained relatively stable. On the other hand, studies using current climate and species distributions to compare species realized niches between native and invasive ranges indicate that niches tend to remain stable during species invasions, but many exceptions (niche shifts) were also reported (Petitpierre et al., 2012; Guisan et al., 2014; Early and Sax, 2014). Additional studies suggest that over spans of decades or a 100 years, species environmental niches rarely change, but as time scales increase to millions of years, niche lability increases (Peterson, 2011).

The temporal niche lability of tree species may be partly due to adaptive evolutionary processes. Genetic modifications may be observed spatially (Heywood, 1991), however, they seldom develop over relatively short periods such as decades while they easily happen throughout several millennia, such as the Quaternary, with distinct and contrasting climate periods (Davis and Shaw, 2001; Davis et al., 2005). Molecular markers from the nuclear and organelle genomes have allowed reconstruction of the evolutionary history of plant species since the last glacial period (Taberlet et al., 1998; Petit et al., 2003). Using neutral markers, phylogeographic studies show that the modern spatial genetic structure over the range of many temperate tree species originated during the past few millennia (Hewitt, 2000, 2004; Petit et al., 2003). These phylogenetic studies show that there are strong interactions between past climate change, changing species ranges, and genetic constitution. While climate variability during the Holocene period was much reduced compared to the climate transitions between glacials and interglacials, species had to migrate and adapt nonetheless during the post-glacial recolonization process, which may have had an impact on their climate requirements and tolerances. The Holocene seems to be an ideal time period for investigating potential temperature shifts of tree species because (1) its reduced climate variability and relative favorability compared to the glacial period allowed taxa to migrate to either track climate changes and potentially to adapt and (2) there are more fossil data-sets for species occurrences and past climates than for older Quaternary time periods.

This study uses observational data at the Holocene time scale to investigate whether or not some European tree species remained within their modern temperature range over the last 10,000 years. Obviously, temperature is only one component of the species niche. However, any shift of the modern range/climate relationship would challenge the assumption of niche conservatism. We examine whether January temperatures experienced by *Abies alba, Fagus sylvatica*, and *Picea abies* are similar to those experienced over approximately 10,000 years during the Holocene. Substantial changes in realized thermal niches over the last several millennia would demonstrate the difficulty of predicting future range shifts solely based on contemporary realized climatic niches and further reflect on the generality of the assumption of niche conservatism.

## Materials and methods

### Quantifying past january temperature

Studies exploring past changes in species realized niches have done so by relating historical species distributions to climate variables obtained from General Circulation Models (GCMs) (e.g., Pearman et al., 2007; Nogués-Bravo et al., 2008; Maiorano et al., 2013). Changes in these relationships have been studied either in time snapshots [usually 6000 and 21,000 years before present (BP)] or in transient simulations since the last glacial period. Notably, the goal of the simulations that produced the employed paleoclimate data was not to determine past climate but rather to examine distinct GCMs under different forcing conditions through comparison with reconstructed paleoclimate from observed data (i.e., Masson et al., 1999). There remains substantial disagreement among paleoclimate simulations and significant biases compared to observations exist, especially for rainfall (Braconnot et al., 2012), that may limit the suitability of these simulations for determining past climatic niches of species.

Here, we use January temperature (Tjan), quantitatively reconstructed over the past 10,000 years in Europe, in recognition of its ecological importance to fulfillment of chilling requirements for budburst and growth (Nienstaedt, 1967; Heide, 1993; Kramer, 1994, 1995; Harrington and Gould, 2015) and the influence of winter temperature on soil temperature, an important factor in determining treeline globally (Körner and Paulsen, 2004). Winter frost may damage needles and wood and, therefore, restrict the range of *A. alba* and *F. sylvatica* in areas where temperature descends below −10°C. *P. abies* is more frost tolerant than *F. sylvatica* and may withstand temperatures down to −17°C. Cold temperatures are a limiting factor for expansion of many tree species in the temperate latitudes, explaining about 80% of the variation in their range sizes (Pither, 2003). Cold temperatures also limit species distributions and diversity along elevation gradients worldwide (Körner and Paulsen, 2004). Furthermore, cold thermal limits of European Holarctic plants may be more conserved than warm limits (Pellissier et al., 2013), thus providing a conservative test for niche changes. Another important abiotic variable for plant species is water availability as influence by precipitation. Palaeoecological studies show that annual precipitation and its seasonal distribution may have complex interactions with temperature in shaping ecosystems over the long term (Cheddadi and Khater, 2016). In the European temperate zone where *P. abies, A. alba*, and *F. sylvatica* occur, temperature is a limiting factor for growth (see i.e., Kramer, 1994 for *F. sylvatica*; Heide (1974) for *P. abies*, and Maxime and Hendrik, 2011 for both *A. alba* and *F. sylvatica*), but precipitation is not. In the present study we have focused on January temperature (Tjan) rather than on precipitation as it has a direct impact on the three focal species and its future increase will probably affect these species more than the expected up to 20% increase of annual precipitation (IPCC, 2014).

Tjan was calculated using fossil pollen records from a network of sediment cores from across Europe, combined with empirical probability density functions of plant taxa identified for the fossil samples, following the method described by Chevalier et al. (2014). Compared to other pollen-based reconstruction methods, this technique avoids the problem related to the lack of analogy between past and modern ecosystems (Jackson and Williams, 2004).

**Figure 1:**
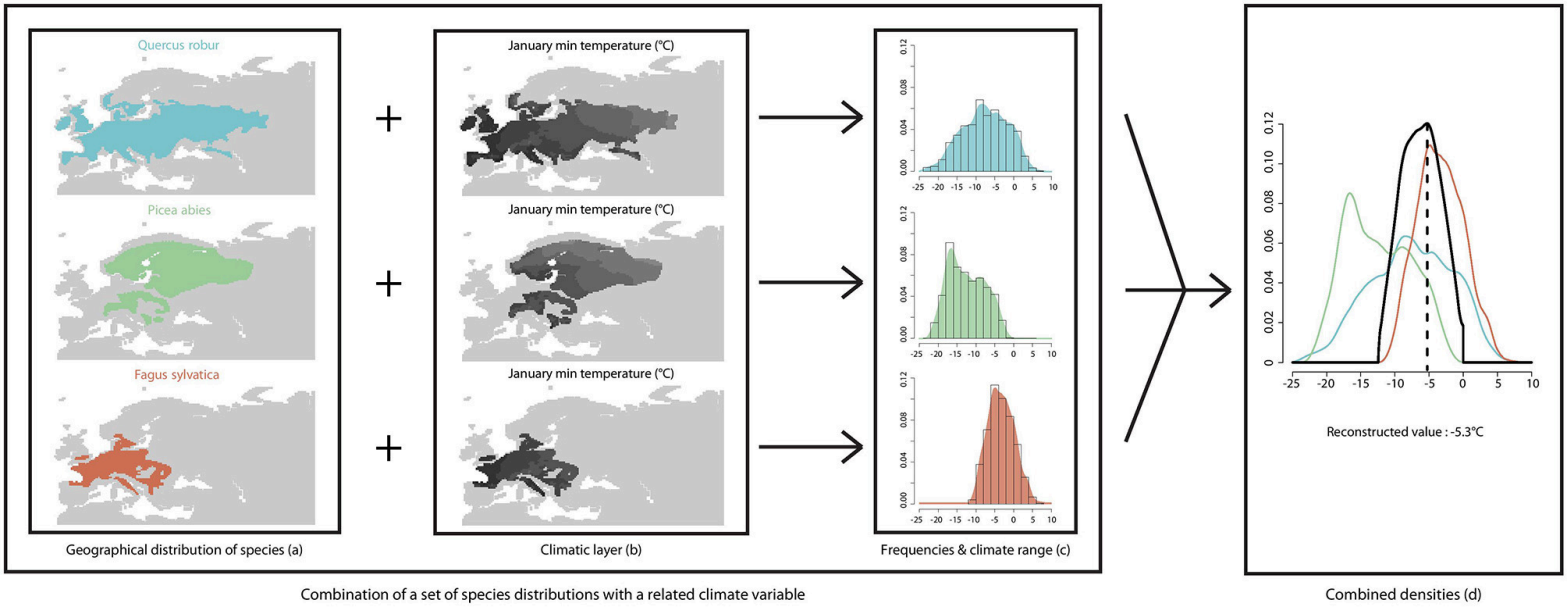
**Schema summarizing the approach used for quantifying a past climate variable from a set of plant species that has been matched to a fossil pollen assemblage**. The modern species ranges are obtained from a plant database **(A)**. The climate variable (January temperature) is obtained from the WorldClim database (Hijmans et al., 2005; **B**). A probability density function (pdf) is used to infer the species-climate relationship **(C)** and the combination of all pdfs provides the intersecting value **(D)**.

We express taxa-climate relationships (Figure 1) as probability density functions (*pdf*, Kühl et al., 2002) that are obtained from modern species distributions (Figure 1A) and values of the focal climate variable (Tjan, Figures 1B,C) in these distributions. Fossil pollen grains are often identified to a genus or a family level which does not allow an accurate identification of the originating species. In order to use the modern species distributions and their related climate, the pdf method requires an assignment of each taxon to a plant species. In the present study we assigned fossil taxa to modern plant species that have the widest climate range and occur in the area. For instance, *Pinus* pollen grains have been assigned to the species *P. sylvestris* whose geographical range encompasses a wide temperature range. *Pinus* pollen grains cannot be identified to the species level which prevents the distinction between temperate and Mediterranean pines. Assigning a pollen grain to a species that has a restricted range (i.e., *P. pinaster* instead of *P. sylvestris*) would bias the climate reconstruction due to an unjustified reduction of the temperature range of the unknown species. Taxa pdfs (Figure 1D) for Tjan were built from a database of 270 georeferenced European plant species from published maps (Jalas and Suominen, 1973; Hultèn and Fries, 1986) and from WorldClim 10' gridded, interpolated weather station data for modern climate (1950–2000 period; Hijmans et al., 2005). The instrumental modern climate data-set used in the present study is set within a warming trend that is related to ongoing anthropogenic climate change (IPCC, 2014). Between years 1861 and 2000 global temperature increased by about 0.6°C (Folland et al., 2001). Over a comparable time period (1901–2000), the winter temperature (DJF) in Europe recorded a warming trend of ~0.08°C per decade (Luterbacher et al., 2004), which is slightly higher than the global value. The human-induced temperature increase recorded over the last century (< 1°C) has already affected the niche of many plant and animal species (Walther et al., 2002). However, this recent temperature increase is lower than the reconstructed amplitude of temperature change over the Holocene (Davis et al., 2003; Cheddadi and Bar-Hen, 2009).

Tjan was calculated from a pollen assemblage of n taxa in a fossil sample “s” as the temperature of maximum probability in the intersection of the n pdfs (Figure 1D), as follows:

$$\begin{array}{l} Tjan\left(s\right)=argmax\left[{\left(\overset{{tax}_{n}}{\underset{{tax}_{1}}{\prod }}pdf{\;}_{tax_{i},Tjan}^{W_{i}\left(s\right)}\right)}^{{\left(\sum W_{i}\left(s\right)\right)}^{-1}}\right] \end{array}$$

where:

*Tjan*(*s*) is the reconstructed climate for sample *s*;

*pdf* is the probability density function of plant *taxon i* (tax_i_) to *taxon n* (tax_n_) for the variable Tjan; n is the number of taxa identified in a sample;

W_i_(*s*) is the weight associated with taxon *i* for sample *s* determined by the pollen percentage of taxon i. Pollen percentages were calculated after removing the pollen counts of the three taxa from the total pollen sum. *argmax* is the function that returns the value for which a function is maximum.

This phenomenological, statistical method does not account for, or depend on, variation in ecological or environmental processes (competition, phenology, dispersal, or evolutionary adaptation, etc.) or their relative importance in structuring paleocommunities. Similarly, estimated variable values do not depend on or account for values that are estimated for preceding periods.

Average Tjan values were calculated for these records to represent fixed time slices every 1000 years (±250 years). Fossil pollen records were selected from the European Pollen Database (EPD, http://europeanpollendatabase.net) for their time resolution and the quality of the associated calibrated age model. Pollen records with less than three radiometric datings or reversed dates were excluded as well as those with a sampling resolution that does not provide a series of at least one sample every 500 years over the covered period of time.

**Figure 2:**
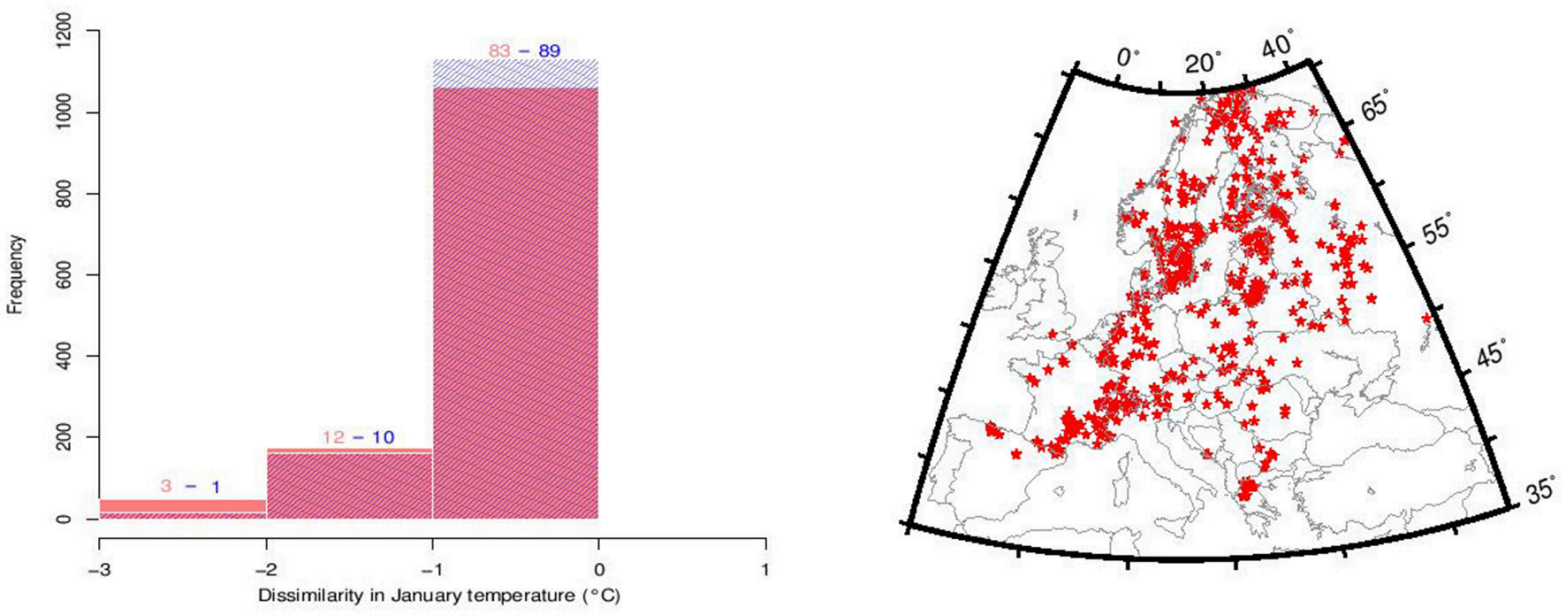
**Evaluation of the impact of *Abies alba*, *Fagus sylvatica*, and *Picea abies* and their Holocene thermal niche shift on the reconstructed Tjan values using dissimilarity between the modern Tjan (WorldClim, Hijmans et al., 2005) and (1) reconstructed Tjan from a data-set of 1132 modern pollen samples from which the three species were excluded (red histograms) and (2) the same modern pollen data-set including the three species but using their overall Holocene Tjan range (blue histograms) based on reconstructed Tjan between 10 and 3 ka (see Figure 3)**. Numbers over the histograms correspond to the percentages of pollen samples that deviate from 0. Between 83 and 89% of the reconstructed Tjan deviate by less than 1°C to the observed Tjan values from WorldClim (Hijmans et al., 2005). The map in the right panel shows the location of the modern pollen samples used for the dissimilarity analysis.

A conceptual difficulty of our climate reconstruction technique lies in its assumption that plant climate envelope is stable, which is what we aim to test. We partially circumvent this issue by excluding observations of the three focal tree species from the data-set used for paleoclimate reconstruction, so that any change in their thermal requirements would not affect the paleoclimate data. In order to evaluate the effect of the exclusion of the three focal taxa, we computed the difference between Tjan reconstructed from a modern pollen data-set and the same pollen data-set excluding the focal three species (Figure 2). In order to estimate the impact of a shift on the reconstructed Tjan, we used the Holocene Tjan range of the three species in the reconstruction procedure and calculated the difference with Tjan based on the full set of species using a modern pollen data-set (Figure 2).

In this preliminary analysis we find that the reconstructed Tjan values remain robust to the exclusion of the three focal tree species from the suite of species used to make the climate reconstruction. Other geological or biological indicators of temperature, such as isotope ratios or chironomid flies, might be considered independent climate proxies, their sparse geographical distributions unfortunately do not allow for spatial climate gridding at the extent of the European continent. Moreover, complete independence of these potential proxies with terrestrial vegetation is unlikely. Similarly, data from GCM simulations of past climates have cryptic dependencies with plant distributions because of the need to specify boundary conditions that depend on vegetation albedo, biomass distribution, and the carbon cycle.

**Figure 3:**
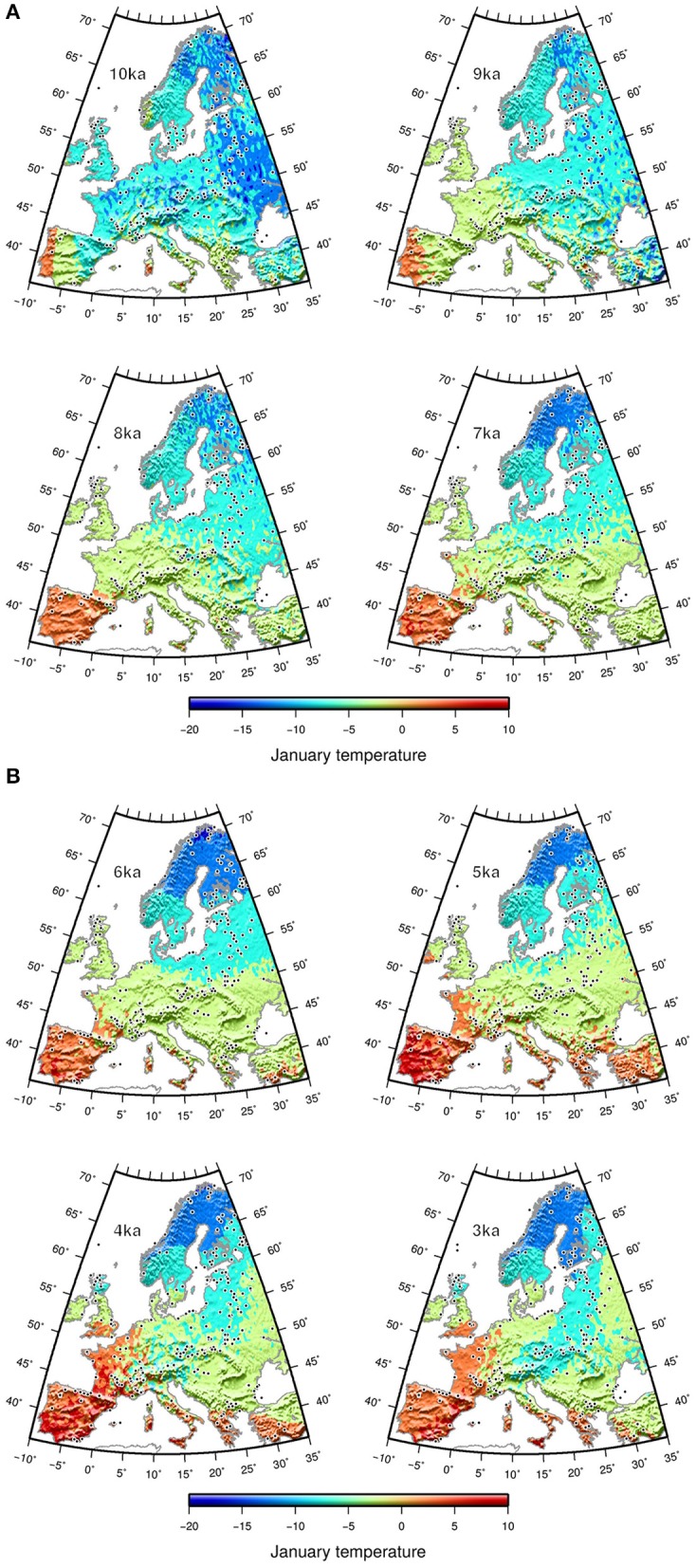
**Interpolated reconstructed mean January temperature (A) from 10 to 7 ka and (B) from 6 to 3 ka from fossil pollen data (black dots)**.

The *Tjan* values obtained in fossil records were spatially interpolated for each time slice (10 to 3 ka) over Europe onto a 0.5° longitude × 0.5° latitude grid using universal kriging (R gstat package, Pebesma, 2004). The interpolated Tjan was then mapped as a smoothed surface using a spherical surface spline in tension (Smith and Wessel, 1990) within the generic mapping tools (GMT, Wessel et al., 2013; Figures 3A,B).

**Figure 4:**
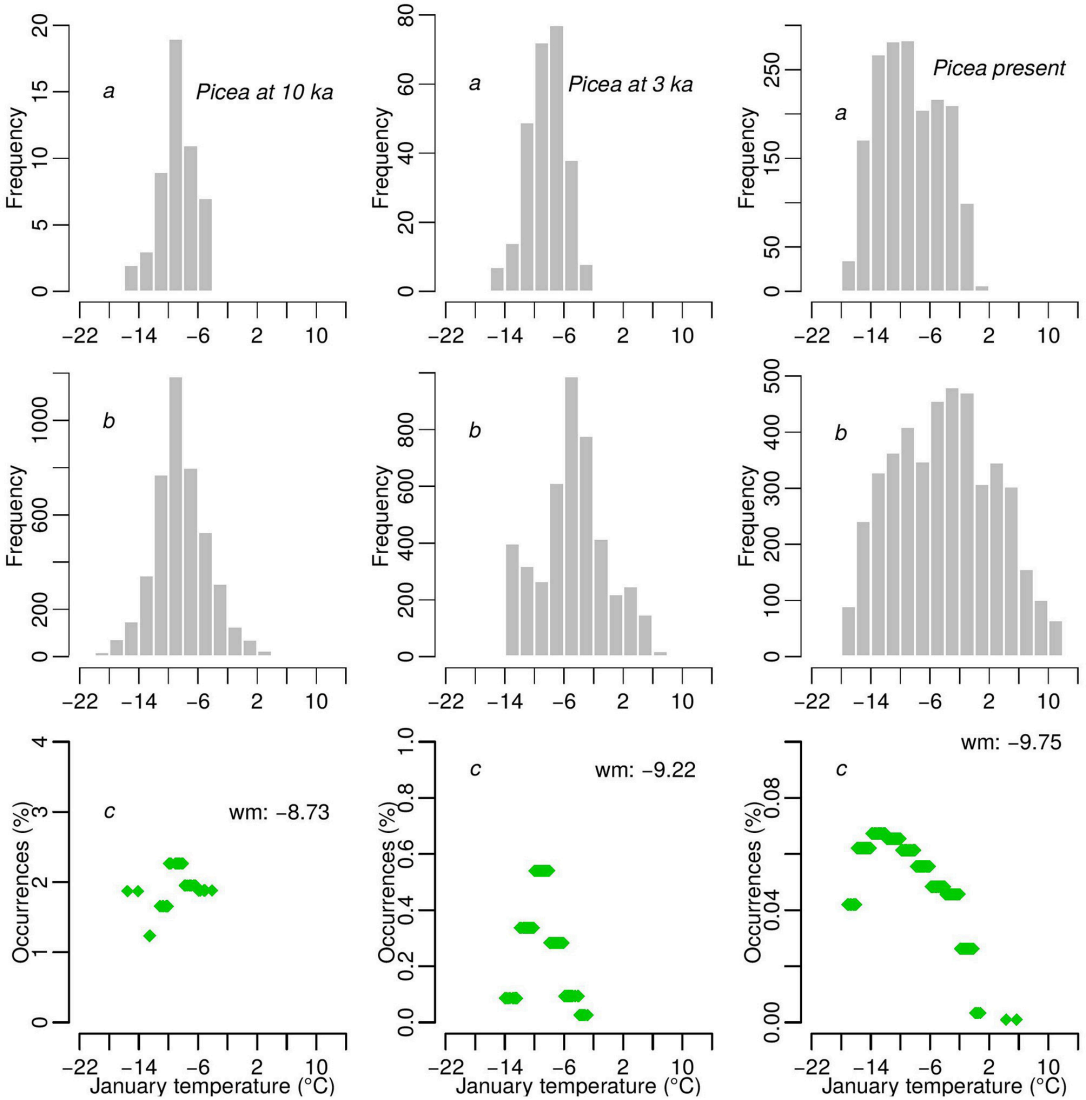
***Picea* as an example of how the reconstructed Tjan values for three time slices (10 ka, 3 ka, and today) were normalized**. The example shows frequency histograms of sites where reconstructed Tjan is obtained **(A)** and the frequency of Tjan gridded values (see Figure 3) over Europe **(B)** within each 2°C bin. The ratios of the two frequencies **(C)** are used to weight Tjan values for each time slice and each species.

The number of time series from which Tjan is obtained increases from 10 to 3 ka, which may potentially affect the comparison of the estimated Tjan range through time. A similar issue has been raised and statistically tackled by weighting the observed temperature values by the availability of climate over a gridded geographical space (Broennimann et al., 2012). The method in the latter work is not directly applicable to our data since these are non-gridded temperature values that are determined by the location of pollen cores and the age of samples. Therefore, we cannot project species range between 10 and 3 ka to a standardized climate space. However, we were able to generate an analogous normalization of the distribution of temperature by weighting each observation by a proportion that represented occupation of similar climate space, which we present for *Picea* at 10 ka, 3 ka, and today as an example (Figure 4). We weighted the reconstructed Tjan values by the relative frequencies of observations in relation to all cores with similar temperature values. Similar temperatures were determined by binning temperature values corresponding to all cores that represented a particular time slice. This normalization still integrates an unavoidable potential bias that is related to the availability of pollen cores in the Tjan gridded space in each bin.

### Recovering past species ranges

We reconstructed the past geographic distributions of *A. alba, F. sylvatica*, and *P. abies* at each time period using macrofossil data and pollen data. These species are ideal case studies because (1) they represent dominant arboreal species in Europe, and (2) they can be identified with a high degree of confidence to species in the fossil record. Unlike many other genera, *Abies, Fagus*, and *Picea* each has one dominant species in Europe, substantially reducing potential errors in the identification of fossil pollen taxa.

We restricted this study to the period prior to 3 ka to avoid human disturbances that strongly impacted European forests in the recent millennia (Kaplan et al., 2009). Therefore, changes observed in distributions of taxa in the past are primarily the result of climate change and ecological processes. The macrofossil data used for identifying species occurrences were obtained from published data-sets (*F. sylvatica*, Magri et al., 2006; *A. alba*, Terhürne-Berson et al., 2004 and *P. abies*, Latałowa and Van Der Knaap, 2006). Occurrences from pollen data were obtained from the EPD using a threshold of pollen percentage of 1% for detection of the three taxa. Since neither macroremains nor pollen of these three taxa were used for paleoclimate reconstructions, we assumed that the reconstructed geographic distributions are independent of the climate variables.

### Recovering january temperature range for each species

**Figure 5:**
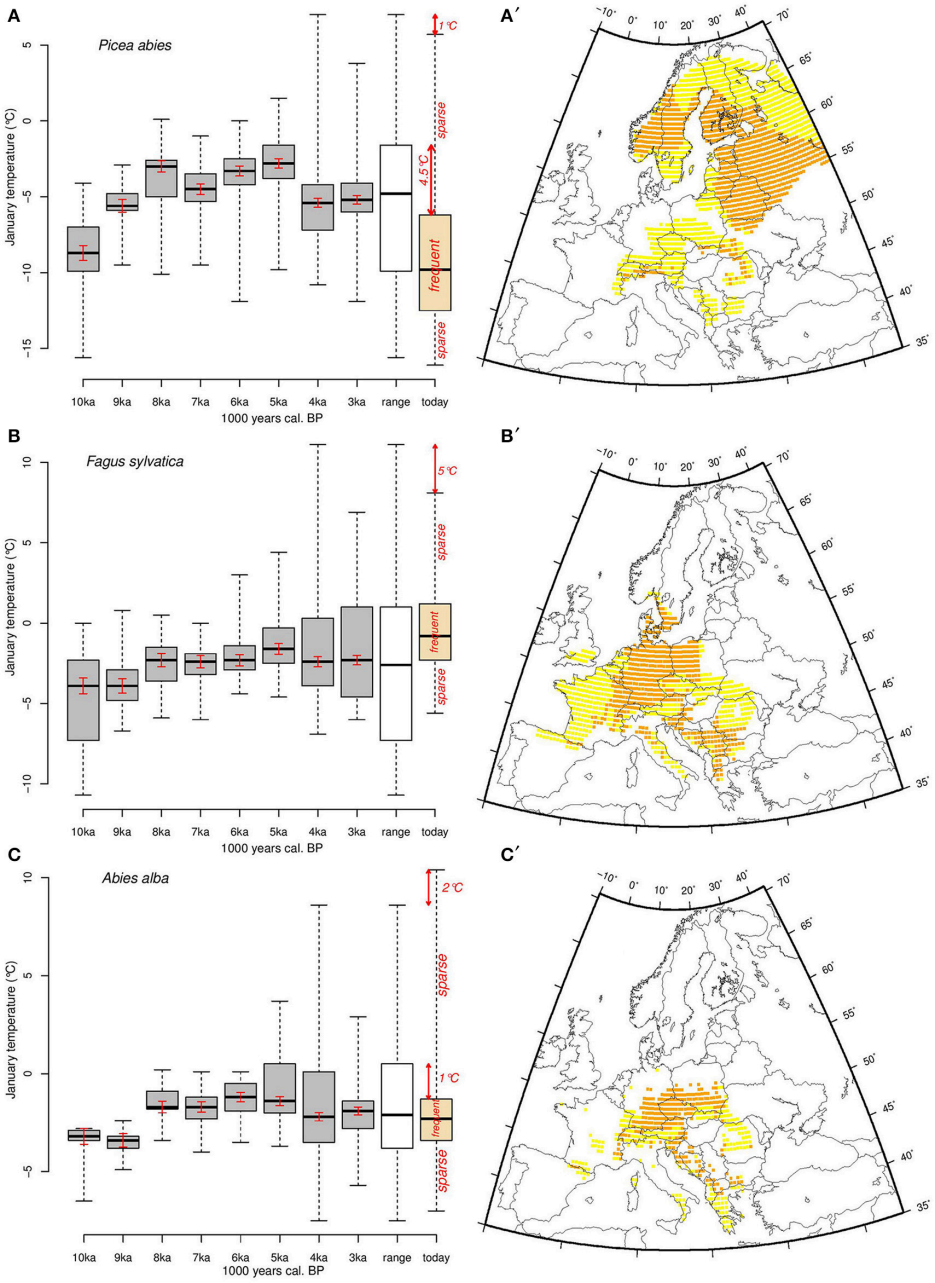
**Thermal amplitude of (A) *Picea abies*, (B) *Fagus sylvatica*, and (C) *Abies alba* for each 1000-year time slice between 10,000 and 3000 years cal**. BP (gray boxplots), the Holocene overall (white boxplots), and the modern (orange boxplots) ranges, respectively. The black line is the median, the boxes represent the first and third quartiles (25 and 75th quartiles, respectively), and the whiskers represent the minimum and the maximum Tjan range. We consider the boxes and the whiskers as the Tjan ranges where populations are frequent and sparse, respectively. Red arrows indicate differences between current limits of Tjan distribution and those over the Holocene. Maps show today's areas of *Picea abies* **(A**′**)**, *Fagus sylvatica* **(B**′**)**, and *Abies alba* **(C**′**)** where Tjan values correspond to the range between the first and third quartiles (orange, corresponding to areas where the species are most abundant) and more extreme values (yellow). The red bars inside the boxes correspond to the estimated uncertainty related to random spatial sampling and to sample size in Monte Carlo simulations.

The extent to which the distribution of Tjan values obtained from fossil data reliably represents the thermal range of a species depends on sample size and geographical distribution. The coring sites are reasonably well distributed over the whole of Europe, but the number of sites where occurrences are observed decreases between 3 and 10 ka. The sample size ranges from 375 at 3 ka for *P. abies*, to 12 at 10 ka for *A. alba* (Table 1) because the geographic distributions of European trees, including the focal three species, were dramatically reduced during glacial times and expanded progressively throughout Europe during the Holocene. As a result, species thermal ranges may be less accurately represented at 10 ka, which corresponds to early post-glacial recolonization. We estimated the uncertainty related to random spatial sampling and to sample size using a Monte Carlo simulation. The modern spatial distribution of the focal species was randomly sampled to extract a Tjan distribution with the same sample sizes as available in the fossil data. Standard errors for Tjan medians were obtained after 1000 iterations and represented as error (Figure 5). The sampling uncertainty associated with Tjan medians was lower than 1°C for the three species at all time periods.

**Table 1:** **Comparison of the medians of the reconstructed Tjan at different time slices in the past (10 to 3 ka and the full range as well) for each species with their modern range**.

**Time slices (ka)**	***Picea abies***				***Fagus sylvatica***				***Abies alba***				**Sites**
	***p*-value**	***p*-value *(w)***	***OCC***	***SE***	***p*-value**	***p*-value *(w)***	***OCC***	***SE***	***p*-value**	***p*-value *(W)***	***OCC***	***SE***	
10	0.86	0.7993	60	0.98	<**10e-05**	<**10e-05**	16	0.98	**0.0033**	**0.0015**	12	0.8	526
9	<**10e-05**	<**10e-05**	113	0.83	<**10e-05**	<**10e-05**	25	0.9	**0.0003**	<**10e-05**	27	0.68	596
8	<**10e-05**	<**10e-05**	155	0.75	<**10e-05**	<**10e-05**	40	0.82	0.3958	0.9483	57	0.59	639
7	<**10e-05**	<**10e-05**	186	0.7	<**10e-05**	<**10e-05**	73	0.76	0.7726	0.8620	93	0.53	671
6	<**10e-05**	<**10e-05**	247	0.66	<**10e-05**	<**10e-05**	110	0.7	**0.0277**	0.0564	110	0.48	706
5	<**10e-05**	<**10e-05**	294	0.62	**0.0012**	0.0019	136	0.66	**0.0027**	**0.0060**	130	0.45	764
4	<**10e-05**	<**10e-05**	335	0.59	<**10e-05**	<**10e-05**	185	0.62	0.2194	<**10e-05**	140	0.42	792
3	<**10e-05**	<**10e-05**	375	0.57	<**10e-05**	<**10e-05**	208	0.59	0.1741	**0.0022**	146	0.4	805
10–3 range	<**10e-05**	<**10e-05**			<**10e-05**	<**10e-05**			0.1995	0.2346			

*We present Wilcoxon tests (p-value) and estimation of the standard error (SE) related to the number of sites where occurrences (OCC) are identified for the three studied species. Since the Tjan medians of all time slices (n = 9) for the three species have been compared to the same one (present), the p-value (α = 0.05) has been adjusted to α/n = 5.6e-3 in accordance with the Bonferroni criterion. The bold p-values are significant at this corrected threshold. P-value and p-value (w) correspond to the Wilcoxon test, with unweighted and weighted Tjan values respectively (see Figure 4)*.

Several issues exist regarding the reconstruction of species past occurrences from fossil data. In general, fossil records allow the identification of *in situ* presence of a species, but tell less about whether the species was effectively absent. Undetected (thus probably small or sparse) populations may occur at a given site but be “silent” in the fossil record (e.g., trees not producing pollen; Hicks, 2006). Also, since tree plant species have different dispersal capacities, long generation time and their propagules may travel considerable distances from parent trees, it is generally accepted that there is “an inevitable time lag between the establishment of seedlings and the maturation of the trees to the stage that they are able to produce pollen” (Hicks, 2006). Reconstructions of broad-scale Holocene vegetation changes, including *Fagus* (Huntley et al., 1989), suggest that the bioclimatic response exhibits only a minor time lag. Although Svenning et al. (2008) argue that the expansion in Europe was delayed by several centuries, Tinner and Lotter (2006) conclude that, due to efficient animal dispersers, the lag was probably of minor importance in the overall postglacial migration process. To address both these issues, we considered that a species was present when it occurred within a broad time window of ± 250 years. In such a window, the potential time lag between the first arrival of many European tree species at a site and the establishment of a substantial population should be barely detectable (Tinner and Lotter, 2006).

## Results

### Reconstructed january temperature

The Tjan values reconstructed in the occupied grid cells yield frequency distributions of Tjan for each species at each time period, allowing us to estimate changes in the thermal range of the three species (Figure 5). Tjan distributions (Figures 5A–C) are generally not normal (Shapiro test, *p* < 0.05) and, thus, they are represented by their medians and the 0.25 and 0.75 quartiles. This interval corresponds to the core range of the species environmental distribution. The significance of differences between past and modern median Tjan values are derived from non-parametric Wilcoxon tests (Table 1).

Discarding the three taxa from the modern plant data-set to quantify Tjan does not affect significantly the reconstructed temperature values (Figure 2). About 83% of the values deviate with less than 1°C, while about 95% deviate less than 2°C in the negative values (Figure 2). More than 99% of the reconstructed positive values deviate by less than 1°C. In a test of temperature reconstruction in the reciprocal direction, use of the Tjan range from the Holocene to quantify modern Tjan values results in 89% of the values deviating less than 1°C and 99% of values less than 2°C when negative (Figure 2). These two dissimilarity tests show (1) that the reconstruction method is robust and, therefore, that the reconstructed Tjan values are reliable and (2) that using a Tjan range of *P. abies, F. sylvatica*, and *A. alba*, as obtained for each species between 10 and 3 ka (Figure 5), to quantify Tjan has a very minor effect on the reconstructed values. This effect impacts mostly, if not exclusively, the reconstructed negative Tjan values.

The interpolated reconstructed *Tjan* values show Tjan ubiquitously lower than 10°C until 7 ka. Early warming (Tjan >0°C) in Europe first took place in the SW part of Europe (Iberian Peninsula), with a gradient of more than 10°C developing toward NE Europe (Figures 3A,B). Subsequently, warmer winters developed over southern Europe, a change corresponding to the onset of “Mediterranean” climate, with dry summers and frost-free winters. Mountainous and other areas lacking pollen data may exhibit stronger interpolation deviation, potentially resulting in low Tjan values, such as those in Sicily and the Peloponnese area at 9 ka. The longitudinal gradient of the Early Holocene switched to a latitudinal gradient after 7 ka and remained so until 3 ka. The Tjan difference between the Mediterranean region and northern Scandinavia after 6 ka was as high as 15–20°C. This latitudinal distribution of Tjan across Europe seems to have persisted until today.

### January temperature range of focal species

The overall *Tjan* range within all the areas recolonized during the Holocene by *P. abies* lies between approximately −16°C and 7°C while the modern range is between −16°C and slightly less than 6°C (Figure 5A). The Tjan at which *P. abies* populations are frequent today (the inter-quartile range) are about 3.5°C colder than at 5 ka, when *P. abies* occupied areas that tended to be relatively warm (Figure 5A). The recolonization of most of Scandinavia by *P. abies* took place after 4 ka (Tollefsrud et al., 2008), which explains the strong shift of the Tjan range toward lower values.

Unlike *Picea*, the main European *Fagus* populations today occupy, on average, warmer areas than at any time during the Holocene (Figure 5B). Nonetheless, half of the frequently occupied temperature range of the modern period overlaps with temperatures also occupied areas in the Holocene. We observe that during the early recolonization process (at 10 and 9 ka), *Fagus* populations occurred in areas with *Tjan* values that were generally lower than at other times during the Holocene. In contrast to *P. abies* and *F. sylvatica, A. alba* occurs in areas that are within its Holocene Tjan range (Figure 5C). Today the geographical range of the species spans a less extended temperature range than during the Holocene.

The Wilcoxon tests (Table 1) show that the medians of *P. abies* and *F. sylvatica* Tjan range differ substantially between modern values and those of the period 10 and 3 ka. This indicates shifts of the thermal range of theses two species between the Holocene and today. The thermal range for *A. alba* was significantly different from that of today during some periods but not in the overall Holocene range (Table 1, Figure 5C).

## Discussion

The use of observed data from fossil records instead of data from model simulations to evaluate the relationship between past species ranges and climate is a challenging issue. First, data on past climate and on species distributions should originate from two independent data-sets and both should have a spatial coverage that minimizes errors of spatial interpretation. Second, the methods for identifying species, recovering their past occurrences and reconstructing their contemporaneous climate values from different proxies should be accurate, reproducible, and have little error. Over the past decades, pollen data have proven to be excellent proxies for the reconstruction of past species distributions (Huntley and Birks, 1983) as well as past climates (Prentice et al., 1991). However, using fossil pollen data to evaluate the relationship between a species occurrence and any climate variable through time requires two important assumptions: First, fossil pollen data represent an image of an ecosystem that is in equilibrium with contemporaneous climate. Second, the observed changes in the past ecosystems are a result of individualistic responses of taxa to climate (Webb, 1986).

Paleoecologists also assume that species evolution is a slow process over the late-Quaternary because fossil data do not provide information on species genetic adaptation to changing climate but rather on species persistence *in situ*, their range expansion/contraction through migration and their extinction (Davis et al., 2005). Fossil pollen assemblages represent a static image of their originating ecosystem, one that prevents evaluating the level of competition between taxa. In the present study, we considered these fossil data as an instantaneous record of that species that occurred in various past ecosystems, each of which occupied portions of a common climate space.

Our results demonstrate that within Holocene ecosystems, the thermal ranges of *P. abies* and *F. sylvatica* differ significantly from the corresponding modern ones. These two species currently occur in areas where Tjan is either warmer (*F. sylvatica*) or cooler (*P. abies*) than the occupied regions of the Holocene. Conversely, *A. alba* is roughly within the same thermal range as during most of the Holocene. The finding that the thermal ranges of two species have varied during the Holocene is partially consistent with previous studies that used modeled Holocene temperatures (Maiorano et al., 2013).

Discrepancies between modeled distributions and ones observed in data on fossil material (e.g., Pearman et al., 2008) may be partially explained by the relationship between the numerical abundance and geographic distributions of species, which is a matter of continuing debate. The traditional view is that species are most abundant near their geographic range center and less so toward their range edge (Brown, 1984). However, when considering the geographic distribution of species based on their environmental niche (i.e., the niche-biotope duality, see Guisan et al., 2014), findings are less conclusive for the hypothesis that species should be most abundant at the center of their environmental distribution (Sexton et al., 2009). Here, in close analogy to abundance, we consider frequency of occurrence along a climate gradient, rather than in geographical space. Thus, we treat the *Tjan* median value of each tree species as the point of greatest frequency of population abundance.

Today *P. abies* is at its greatest abundance in areas where *Tjan* is between ca. −12 and −6°C (1st and 3rd quartiles, Figures 5A,A'). Assuming adequate plasticity and genetic variation, populations in these areas may potentially withstand a *Tjan* increase of about 4.5°C *in situ* (Figure 5A), while a similar temperature increase may have greater effects on populations that grow at the upper *Tjan* limit (mainly from Romania to the Dalmatian coast). The 1°C difference between the Holocene ranges and the modern one is not significant if we take into account the standard error related to the potential bias linked to the distribution of past species occurrences (Table 1 and Figure 5). However, an increase of Tjan may have greater effects on *Picea* populations at the warm (southern) edge of the species range than on climatically central populations.

*Fagus* populations are frequent nowadays in areas where *Tjan* is between ca. −2.5 and 1.5°C (inter-quartile range, Figures 5B,B'), but currently occur infrequently in areas with *Tjan* values that are 5°C lower than those observed during the Holocene (Figure 5B). Unlike *P. abies, F. sylvatica* populations frequently occur today in areas that have *Tjan* values that are as high as those of the Holocene. Such thermal range occupancy suggests that, where it is frequent today, *Fagus* will likely be threatened by increasing temperature, while the sparse populations that occur at the cold edge of the distribution may withstand an increase of *Tjan* of up to 5°C. *F. sylvatica* is more sensitive to cold winter temperature and spring frost than *P. abies*, so climate warming may promote its expansion into areas where the range of the two taxa overlap, such as southern Sweden and/or potentially in the mid to high elevations in Poland and the Carpathians. Simulations for the next century show that *F. sylvatica* may have to migrate further north while experiencing a loss of up to 29% of its current habitat (Kramer et al., 2010). This projection may well be valid since populations are most frequently located in areas that are at the highest Tjan values observed in the Holocene thermal range (Figure 5B). Furthermore, the species migration rate (100–250 m.yr^−1^) estimated by Tinner and Lotter (2006) is lower than the velocity of the expected temperature change (~800 m.yr-1) for the temperate deciduous biome (Loarie et al., 2009). Nonetheless, the magnitudes of these velocities are highly dependent on the resolution of the underlying climate data-sets (Dobrowski et al., 2013).

The patterns shown by *A. alba* differ from those of *P. abies* and *F. sylvatica*. Unlike the other two species, the full modern *Tjan* range of *A. alba* is at its greatest extent since the early Holocene (Figure 5C). However, the most frequent populations occupy a much narrower *Tjan* range (ca. −3.5 to −1.5°C, Figures 5C,C') than during the Holocene (ca. −4 to +0.5°C), which suggests these environmentally central populations may be able to withstand a somewhat wider temperature range than they currently experience (Figure 5C). Conversely, there are sparse *A. alba* populations today that experience *Tjan* as high as +10°C, which is about 2°C higher than for any known Holocene population (Figure 5C). Thus, the modern populations at the warmer end of the species distribution (Mediterranean mountains) may be threatened by further climate warming. While ecological niche models suggest that *A. alba* currently occupies less than 50% of its potential range (Svenning and Skov, 2004), our results suggest, in contrast, that this species currently occupies its full thermal range, and that the distribution of the populations within that thermal range may change as *Tjan* increases. This may occur through latitudinal migration, simple expansion of population density within other areas of the modern distribution (but without any further geographical expansion; see Figure 1E in Guisan et al., 2014), or expansion to fill sparsely and unevenly populated areas. The discrepancy with Svenning and Skov (2004) may be related to potential deviations during the interpolation of their climate data set, or the particular modeling algorithm they employ.

Ecological modelers and palaeoecologists both aim to address a crucial question: will major tree species withstand on-going global warming *in situ* (because they can tolerate a wider temperature range than what they currently encounter), migrate to areas that acquire suitable conditions, or simply decline. Species modeling approaches suggest that the impact of future climate change will be substantial in Europe (Thuiller et al., 2005). On the other hand, some fossil-based distribution data, such as for *A. alba*, suggest little risk will arise with climate warming (Tinner et al., 2013). In contrast, a recent palaeoecological study (Seppä et al., 2015) shows that the range of hazel (*Corylus avellana*) previously expanded about 4° northward from its current geographical limit in Fennoscandia as a response to a temperature increase of 2.5°C during the mid-Holocene, constituting an increase similar to some projections for response to on-going climate change. Species responses to climate warming may vary substantially and, thus, require focused study. While we concentrated on three tree species because of the direct correspondence between fossil pollen taxa (identified to genus) and species-level identification (e.g., compared to the 22 European species *Quercus* that produce indistinguishable pollen), one might expect that the thermal ranges of additional tree species may have been different in the past. Additional gridded proxy data, other than pollen, as well as identification of fossil taxa using ancient DNA (Parducci and Petit, 2004) will be needed to address this issue.

Evaluating the climatic limits of species distributions through time is a key issue to understanding species responses to future climate change. Extended data-sets, such as those arising from fossil pollen, are sufficient for reconstructing past geographical distributions of many taxa and for estimating important climate variables. However, they do not contain any information about the adaptive evolution of species over the period of interest (i.e., the Holocene). When assessing the relationship between past species distributions and climate change, paleoecologists tend to assume that ecological responses, such as range shifts, migration rates, and recolonization, are the dominant processes that impact observed species distributions (Huntley et al., 1989; Huntley and Webb, 1989). However, other biotic responses such as phenotypic plasticity and genetic adaptation were also important during the Quaternary (Davis and Shaw, 2001). While evolutionary rates are likely slower than rates of ecological response to climate change, at least during the late Quaternary, promising developments in ancient DNA technology (Giguet-Covex et al., 2014) may help to determine whether or not species genetically adapted to past climate change.

Fossil records may indicate on-going and successful accommodation to climate when a species range remains in dynamic equilibrium with temperature over time (at least during the late-Quaternary period; Davis and Shaw, 2001). The assumption that species distributions in the past (or today) are at equilibrium with their contemporary climate has yet to be demonstrated. If species are not at equilibrium with climate, then there may be little justification to expect stasis in realized niches through space and time (Araújo and Peterson, 2012). Based on observed data, Araújo and Pearson (2005) suggest that assemblages of plants and breeding birds are closer to equilibrium with climate than other organisms and, therefore, their future ranges may be well predicted by niche-based models. Recent work based on fossil data, however, suggests significant potential disequilibrium between vegetation and climate at both leading and trailing edges of species ranges (Svenning and Sandel, 2013). This apparent disequilibrium arises in part from the estimated time lag of the response of individual species ranges to post-glacial warming (Webb, 1986). Thus, strong differences among species in the degree of distribution equilibrium may occur due to varying dispersal abilities, competition with other species, soil conditions, and other factors that may affect the ability of species distributions to track changing climate.

## Conclusions

Modeling approaches are necessary to project how species distributions could change in the future. Here we show that fossil data provide valuable information on how temperature changes might affect different parts of the range of a species in different ways. We demonstrate how fossil data can complement data on contemporary species distributions to provide valuable information on species tolerances to climate variation. Our results reveal changes in the thermal ranges of the three species with respect to an influential climate variable over the past 10,000 years. The results further suggest that differing impacts of future climate warming, depending on the density (sparsity) of populations. In parallel with approaches based on climate models, data on past species occurrences and reconstructed climates derived from them can provide independent estimates of whether species can cope with climate change and, potentially, what parts of their distributions will be the most threatened.

Nevertheless, the past reconstructions need to be constrained by an evaluation of the infra-specific adaptive ability of species, their dispersal capacity, their interspecific competitive ability, and the ecophysiological relationship of each species to different climate variables. Moreover, one climatic variable such as Tjan, may be considered as restrictive for evaluating the ranges of other species than our focal species. Therefore, other additional climate variables are necessary to better define the climatic niche of most plant species and their potential adaptive capacity through time. Water availability, through the annual amount of precipitation and its seasonal distribution, is a key variable for species spread, their persistence and populations expansion. Without an evaluation of these variables, the question remains as to whether a species is capable of withstanding a wider climatic range than that occupied currently and, thus, has portions of the full thermal range that remain unoccupied.

## Author contributions

RC performed all data analysis, programming, computational work, figures, and tables and has written the original manuscript. PBP has improved several versions of the manuscript and contributed substantially, in a decisive way to its final version. MCa contributed to improving many statistical aspects of the study and particularly to estimating the errors through the Monte-Carlo simulations. MCh contributed to improving the climate reconstruction method. All co-authors have contributed to designing the study and refining its objectives in the frame of the EU ECOCHANGE project. MA, LM, ME, AG, MCa, and PBP have contributed to improving the manuscript at different stages.

### Conflict of interest statement

The authors declare that the research was conducted in the absence of any commercial or financial relationships that could be construed as a potential conflict of interest.
